# How Capsular Exopolysaccharides Affect Cell Surface Properties of Lactic Acid Bacteria

**DOI:** 10.3390/microorganisms8121904

**Published:** 2020-11-30

**Authors:** Carsten Nachtigall, Cordula Vogel, Harald Rohm, Doris Jaros

**Affiliations:** 1 Institute of Natural Materials Technology, Technische Universität Dresden, 01062 Dresden, Germany; harald.rohm@tu-dresden.de (H.R.); doris.jaros@tu-dresden.de (D.J.); 2 Institute of Soil Science and Site Ecology, Technische Universität Dresden, 01062 Dresden, Germany; cordula.vogel@tu-dresden.de

**Keywords:** capsular exopolysaccharides, cell surface hydrophobicity, SEM, lactic acid bacteria, microbial adhesion to hydrocarbons (MATH), microscopy, moisture load, ruthenium tetroxide, *Streptococcus thermophilus*, ultrasound, *Weissella cibaria*

## Abstract

Some lactic acid bacteria are able to produce exopolysaccharides that, based on localization, can be distinguished in free and capsular or cell-bound exopolysaccharides (CPS). Up to now, the former were the focus of current research, mainly because of the technofunctional benefits they exhibit on fermented dairy products. On the other hand, CPS affect the surface properties of bacteria cells and thus also the textural properties of fermented foods, but data are very scarce. As the cell surface properties are strongly strain dependent, we present a new approach to investigate the impact of CPS on cell surface hydrophobicity and moisture load. CPS positive and negative *Streptococcus thermophilus* and *Weissella cibaria* were subjected to ultrasonication suitable to detach CPS without cell damage. The success of the method was verified by scanning electron and light microscopy as well as by cultivation experiments. Before applying ultrasonication cells with CPS exhibiting an increased hydrophilic character, enhanced moisture load, and faster water adsorption compared to the cells after CPS removal, emphasizing the importance of CPS on the textural properties of fermented products. The ultrasonic treatment did not alter the cell surface properties of the CPS negative strains.

## 1. Introduction

The formation of exopolysaccharides is a widespread ability amongst lactic acid bacteria and is closely associated with texture-enhancing effects in fermented dairy products [1,2]. Gram-positive bacteria such as Streptococcus (hetero-fermentative) or Weissella (homo-fermentative) are able to produce exopolysaccharides that are then freely present in the fermentation medium. The underlying synthesis mechanisms, however, differ between the genera: while hetero-polysaccharides by *Streptococcus thermophilus* are synthesized inside the cell by the action of different glycosyltransferases (Wzx/Wzy-dependent pathway), homo-polysaccharides from *Weissella cibaria* (glucans or fructans) are produced extracellularly by the action of a single sucrase from sucrose as the only substrate [3,4,5]. In particular, hetero-polysaccharides exhibit a large variety in structure due to differences in monosaccharide composition (mainly glucose, galactose, rhamnose) and in their anomeric configuration, and in regard to functional groups, linkage type, branching, and side chain length [6]. Homo-polysaccharides mainly differ in their glycosidic linkages and degree of branching [7]. Improved water binding capacity, viscosity enhancement, and reduced syneresis of fermented products are only a few factors that have raised interest in microbial, in situ produced free exopolysaccharides and their possible ex situ application such as thickening agents or stabilizers in non-fermented foods [8,9,10,11].

In addition to the well-studied free exopolysaccharides, some strains are also able to synthesize exopolysaccharides that remain covalently attached to N-acetylmuramic acid residues of the cell wall peptidoglycan; these are usually denoted as capsular or cell-bound exopolysaccharides (CPS) [5,11,12,13]. Generally, it is assumed that CPS contribute to adhesion, for instance, on solid surfaces or in food matrices, and to the associated formation of biofilms [14,15], to adhesion in the intestinal tract [16], or to stress protection (e.g., salt and acid stress) [17], and therefore affect the cell surface properties and interactions with the surrounding matrix [18]. A few studies have pointed out the improved properties of fermented dairy products, for example, increased water retention capacity and melting behavior of mozzarella with CPS forming *S. thermophilus* [19,20] or of cheese with *Lactobacillus helveticus* or *Lactococcus lactis* [21,22,23]. Improved creaminess, enhanced textural properties and viscosity [24], and higher gel stiffness [25] were observed in acid gels. Hassan and Frank [26] reported that CPS improved the adhesion of *E. coli* cells to apple and lettuce surfaces, but no differences in electrostatic and hydrophobic interactions were found compared to CPS negative cells.

CPS are also important concerning the virulence of pathogenic microorganisms such as *Erwinia stewartii*, *Erwinia amylovora* or *Xanthomonas campestris* (corn pathogens) [27,28], *Lactococcus garvieae* (fish pathogen) [29], and *E. coli* K4, *S. pneumoniae*, or *Salmonella enteriditis* (human pathogens) [30,31], but the underlying mechanism is not always fully understood. In the case of salmonella, the presence of CPS induces a resistance of the cell surface against acids and bleaching agents [31]. The antibiotic resistance of *S. pneumoniae* can also be retraced to its CPS as antibiotics induce CPS to change the serotype by genetic recombination [32,33]. However, anti-CPS antibodies may be able to defend the host organism against pathogenic encapsulated bacteria [28]. Common measures for the characterization of the bacteria surface include cell surface hydrophobicity (CSH) as determined by the microbial adhesion to hydrocarbons (MATH) assay or by contact angle measurements, the zeta potential, or the moisture load [15,34,35]. Positive correlations were found between CSH and autoaggregation properties of propionibacteria [36], and between CSH and protein content of the cell surface of *Lactobacillus rhamnosus* [37]. The presence of polysaccharides at the cell surface resulted in a more hydrophilic character and lower CSH values [17,38,39]. In turn, cell surfaces without CPS showed higher CSH due to the increased amount of nonpolar protein side chains [11]. However, it has also been shown that CSH is variable over a wide range within genera and strongly strain-dependent. Furthermore, it depends on cultivation conditions such as medium composition or temperature and the state of cultivation [40,41,42,43,44]. This resulted in CSH values for *S. thermophilus* between 0.05 and 98%, but the influence of CPS on CSH was not investigated [45,46,47].

It is known that CPS exhibit a significant impact on the texture properties of fermented foods, but, up to now, the influence of CPS on the cell surface and therefore on interactions in fermented products has only been studied by comparing CPS positive and negative strains [11,17]. This may be, however, criticized because of the strong strain dependency of the cell surface properties. This is why we followed a different approach in this study. The aim was to investigate the impact of CPS on cell surface hydrophobicity and moisture load before and after CPS detachment of different *S. thermophilus* and *W. cibaria* strains. For CPS removal from the cells, an ultrasonic treatment was used, and the ultrasound parameters (amplitude, sonication time) were chosen in a way that it did not affect the cell viability, which was verified by imaging methods and cultivation experiments.

## 2. Materials and Methods

### 2.1. Materials

*S. thermophilus* ST143+ and ST143− were provided by Christian Hansen A/S (Hørsholm, Denmark), *S. thermophilus* DGCC7984 by Danisco Deutschland GmbH (Niebüll, Germany), and *W. cibaria* DSM14295 by Deutsche Sammlung von Mikroorganismen und Zellkulturen GmbH (Braunschweig, Germany). All strains were stored as cryocultures at −80 °C. Tryptone and n-hexadecane were purchased from Carl Roth GmbH & Co. KG (Karlsruhe, Germany) and stored under oxygen-free conditions. Whey permeate powder was obtained by Wheyco GmbH (Altentreptow, Germany) and ruthenium tetroxide (RuO_4_, 0.5% stabilized aqueous solution) by Polysciences Inc. (Warrington, FL, USA).

### 2.2. Cultivation Conditions

Cultivation of *S. thermophilus* was carried out in an enriched whey permeate medium, consisting of 60.0 g/L whey permeate powder and 10.0 g/L tryptone. *W. cibaria* was cultivated in whey permeate medium (100 g/L) enriched with 10.0 g/L tryptone, 2 g/L ammonium sulphate, 9.9 g/L glucose monohydrate, and 34.2 g/L sucrose. After inoculation, the pre-cultures were incubated for 24 h at 40 °C (*S. thermophilus*) or 37 °C (*W. cibaria*) under anaerobic conditions (10% H_2_; 10% CO_2_; 80% N_2_). A total of 50 mL fresh medium was inoculated with 1% (*v*/*v*) pre-culture and incubated under anaerobic conditions for 16 h at 40 °C (*S. thermophilus*) or for 24 h at 25 °C or 30 °C (*W. cibaria*). The fermentation broth was then used immediately for further investigations or stored at −80 °C. All cultivations were performed in duplicate.

### 2.3. Optical Density of Cell Suspensions

The optical density OD_577_ [−] was determined by measuring the turbidity of the cell suspensions at 577 nm. For OD_577_ >0.4 the suspensions were diluted, and turbidity was multiplied with the dilution factor. Cell-free supernatants, obtained by centrifugation (14,100× *g*; 5 min; room temperature), were used as blanks.

### 2.4. Determination of Bacteria Cell Chain Length

The length of the *S. thermophilus* cell chains was derived from microscopic images (Axiostar microscope, Carl Zeiss Microscopy GmbH, Jena, Germany) at 1000-fold magnification with at least 200 cell chains per sample for statistical investigation. The images were processed with Fiji [48] and the resulting cell chain length distribution characterized by theoretical x_10_, x_50_ (median) and x_90_ values.

### 2.5. Ultrasonication of Bacteria Cells

To remove CPS from the bacteria cells, a 2 mL cell suspension was treated with a UDS751 ultrasound disintegrator (Topas GmbH, Dresden, Germany), operating at 24 kHz and at an amplitude of 100% for 18 s unless noted otherwise. During ultrasonication, the samples were cooled in an ice bath.

### 2.6. Visualization of Capsular Exopolysaccharides

#### 2.6.1. Light Microscopy

The presence of CPS was visualized by negative ink staining [49]. Four µL of cell suspension was mixed with 6 µL India ink on a glass slide and covered with a cover slip. Microscopic images were taken at 1000-fold magnification. As the ink is not able to permeate into the polysaccharide layer, CPS occur as a white zone around the cells.

#### 2.6.2. Scanning Electron Microscopy (SEM)

To remove disturbing matrix substances, 2 mL of cell suspension was centrifuged (14,100× *g*; 5 min; room temperature) and washed three times with 9.0 g/L sodium chloride solution, and finally with deionized water. The cell pellets were fixated, stained, and freeze-dried for high-resolution images. Briefly, samples were fixed in 40 g/L paraformaldehyde with 2% (*v*/*v*) glutaraldehyde in 0.1 mol/L sodium cacodylate buffer for 45 min. Subsequently, the samples were washed three times with 0.1 mol/L sodium cacodylate buffer for 10 min, and then stained with 0.5% RuO_4_. The residual RuO_4_ was removed by washing five times in deionized water. After fixation and staining, the samples were frozen and subsequently freeze-dried. The dry cell material was fixed on aluminum specimen holders using conductive double-sided adhesive carbon tabs and coated with approximately 40 nm carbon, using an EMITECH K905 Carbon Coater (Emitech Ltd., Ashford, Kent, England). This was done to minimize charging effects and to allow better resolution, maintained under high vacuum.

Bacteria cells before and after ultrasonication were visualized with an environmental scanning electron microscope under high vacuum (Quanta FEG 650, Thermo Fisher Scientific, Waltham, MA, USA). Several images were gathered from each sample at 10 kV beam energy and a spot size of 2.5. The cell diameters of approx. 50 cells per sample were determined from SEM images using Fiji [48].

### 2.7. Cell Surface Properties

#### 2.7.1. Cell Surface Hydrophobicity

Cell surface hydrophobicity was determined with the MATH test according to Rosenberg et al. [50] with some modifications [49]. Approx. 5 mL of a fresh cultured cell suspension were centrifuged (19,000× *g*; 15 min; 4 °C) and the cell pellet washed twice with 9.0 g/L sodium chloride solution. Subsequently, the cells were resuspended in phosphate buffered saline (PBS) buffer (0.20 g/L potassium chloride; 8.00 g/L sodium chloride; 1.44 g/L disodium hydrogen phosphate; 0.24 g/L potassium dihydrogen phosphate; pH 7.4) and OD_577_ of the suspension adjusted to 0.4 (A_0_ [−]). Three mL of suspension was transferred into glass test tubes, washed with 37% (*v*/*v*) hydrochloric acid for 10 min in an ultrasonic bath prior to use, and mixed thoroughly for 60 s with 0.3 mL n-hexadecane. As a blank, 3.0 mL of PBS buffer instead of cell suspension was used. The mixture was transferred into disposable polystyrene cuvettes and placed on a magnetic stirrer (Cimarec i Multipoint 15, Thermo Scientific, Waltham, MA, USA) for phase separation (15 min, 25 °C). The rotation speed was adjusted to prevent cell sedimentation, but without impeding phase separation. After 15 min, the OD_577_ of the aqueous phase was read and designed as A_1_ [−] or A_blank_ [−]. All determinations were performed in triplicate. CSH [%] was calculated using Equation (1):

(1) $$\mathrm{CSH}=\frac{A_{0}-(A_{1}-A_{\mathrm{blank}})}{A_{0}}\;\times \;100$$

#### 2.7.2. Moisture Load

Cell suspensions were washed three times with deionized water to remove low-molecular substances, followed by freeze-drying. The moisture-free samples were stored in a desiccator over P_2_O_5_ until further use.

Moisture load was determined as described previously with some modifications [51]. An approx. 5 mg sample was placed in a quartz crucible of a Q5000SA dynamic vapor sorption analyzer (TA instruments, New Castle, DE, USA). Relative humidity (RH) in the measuring chamber was set to 0% and incrementally increased to 98% (steps of 10%, last step 8%) unless noted otherwise. The mass was read throughout the analysis by the instrument’s microbalance. Once one of the abort criteria (I) constant mass (≤0.01% within 5 min) or (II) time limit of 500 min without achieving equilibrium were met, the next RH condition was applied. The moisture load X [g H_2_O/g dry mass] at each RH level was calculated from the final equilibrium mass of each step. For a relative humidity of 0–80% and the respective water activities of 0.0 ≤ a_w_ ≤ 0.8 (0% < RH < 80%), X was fitted into the Guggenheim–Anderson–De Boer (GAB) equation [52,53]:

(2) $$X=\frac{X_{0}\cdot C_{\mathrm{GAB}}\cdot k_{\mathrm{GAB}}\cdot a_{W}}{\left(1-k_{\mathrm{GAB}}\cdot a_{W})(1-k_{\mathrm{GAB}}\cdot a_{W}+C_{\mathrm{GAB}}\cdot k_{\mathrm{GAB}}\cdot a_{W}\right)}$$

### 2.8. Statistics

All results are displayed as arithmetic mean ± standard deviation. Differences were statistically tested with the multiple Tukey–Kramer test (*p* < 0.05) after performing one-way analysis of variance (ANOVA) using SPSS Statistics 25 (IBM, New York, NY, USA).

## 3. Results and Discussion

### 3.1. Visualization of Capsular Exopolysaccharides

**Figure 1:**
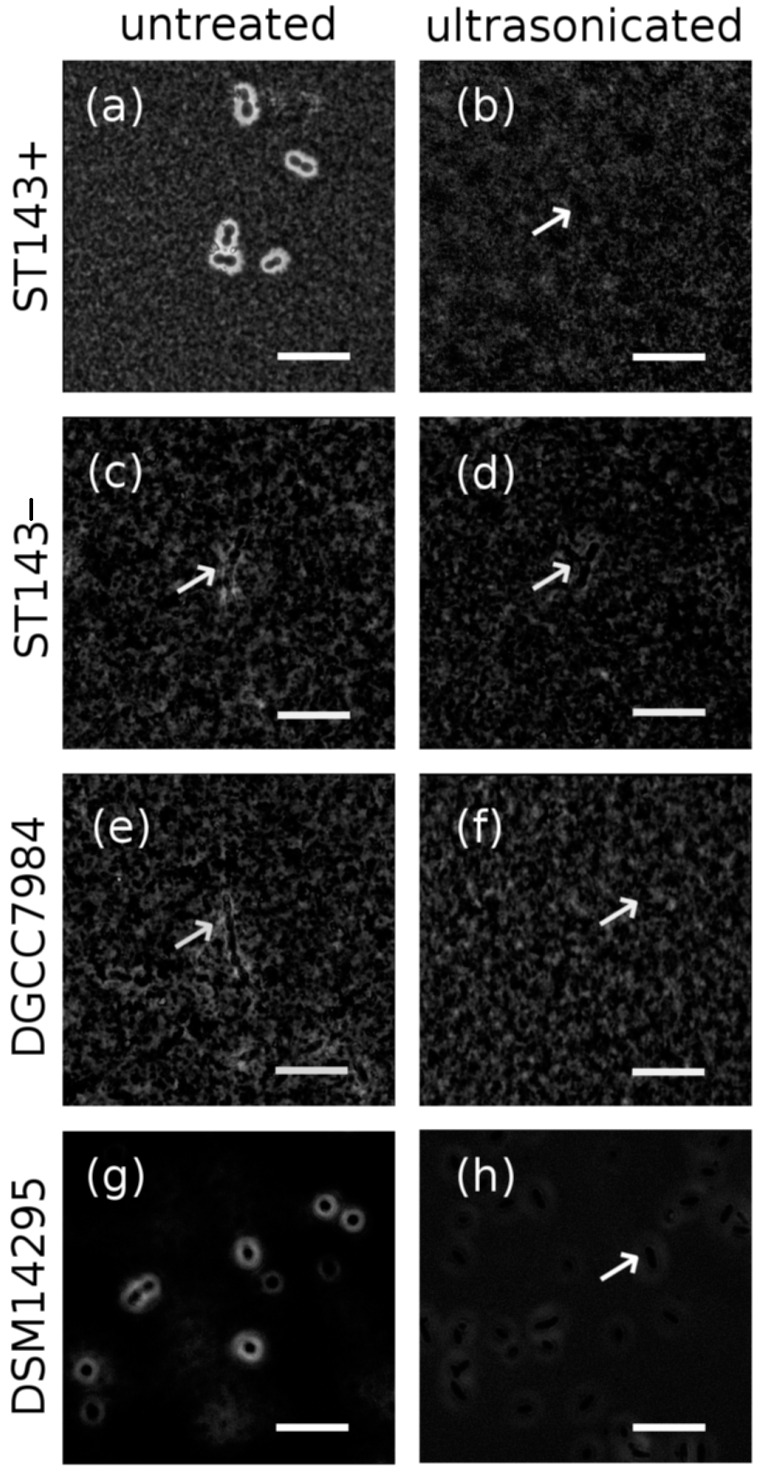
Microscopic images of the cell suspensions of *S. thermophilus* ST143+ (**a**,**b**), ST143− (**c**,**d**), DGCC7984 (**e**,**f**), and *W. cibaria* DSM14295 (**g**,**h**) stained with India ink for CPS visualization, before (left) and after (right) ultrasonic treatment for CPS removal. Magnification: 1000×, scale bar: 10 µm. Arrows indicate microbial cell chains.

After contrast staining, *S. thermophilus* ST143+ and *W. cibaria* DSM14295 showed white zones around the cell chains and were thus classified as CPS producers (Figure 1a,c,e,g). *S. thermophilus* ST143− and DGCC7984 did not produce CPS and were used as negative controls.

Obtaining sufficient contrast is a challenge for the visualization of bacterial features by SEM. A poor contrast frequently arises from phases providing only small differences in electron density. This difficulty can be overcome by staining the hydrocarbon polymer phase with a heavy-atom-containing reagent such as osmium tetroxide (OsO_4_) or ruthenium tetroxide (RuO_4_), which we chose because of its lower toxicity [54]. In addition, RuO_4_ is supposed to react strongly with polar lipids, proteins, glycogen, and monosaccharides [55]. Joubert et al. [56] applied RuO_4_ for staining *Aspergillus fumigatus* biofilms and described it as an excellent contrasting agent for cellular and extracellular matrix components of the biofilm whereby, in contrast to OsO_4_, fibrous ultrastructures were resolved both on and between hyphae.

**Figure 2:**
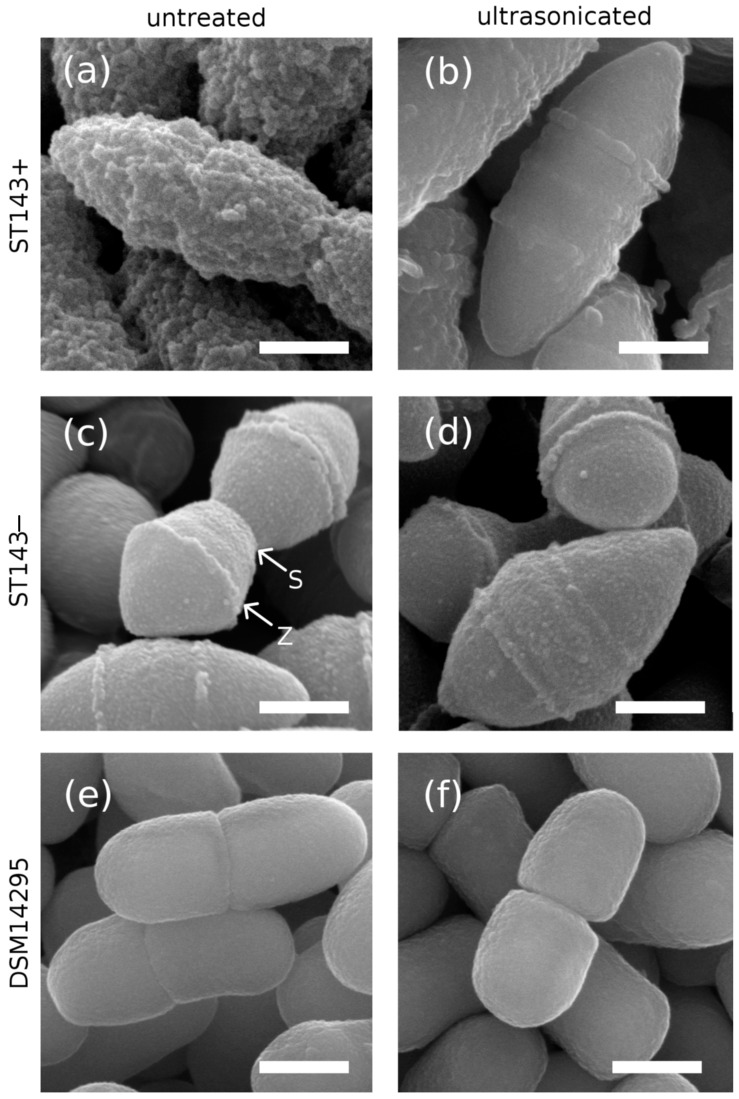
Comparison of the cells of *S. thermophilus* ST143+ (**a**,**b**), ST143− (**c**,**d**), and *W. cibaria* DSM14295 (**e**,**f**) before (left) and after (right) ultrasonic treatment for CPS removal as demonstrated by SEM images. Z: Z-ring; S: septum; scale bar: 500 nm.

*S. thermophilus* ST143+ showed typical elongated ellipsoid-shaped cells, with the cover of CPS causing a rough, rugged surface (Figure 2a). The single cell chains tended to associate and aggregate as was also observed for CPS-producing *Propionibacterium freudenreichii* [57,58]. Structural features attributable to cell division were not visible because they were superimposed by the CPS layer. The free exopolysaccharides from ST143+ consist of a pentameric repeating unit build of 1,4-linked α-/β-glucose and β-galactose with 1,6-linked dimeric side chains [59]. We assumed that the CPS had the same structure as their synthesis pathway did not differ from that of the free exopolysaccharides. However, it is still not possible to predict the functionality (e.g., water binding, texture enhancement) of a specific EPS only from its structure. This is also true for CPS-covered cells in fermented foods [28].

As ST143− does not produce any exopolysaccharides, its cell surface appeared smooth. Division septa (S) and Z-rings (Z) are proteinous structures responsible for cell division [60,61], and were clearly visible depending on the individual state of cell division (Figure 2c). Streptococci and Weissella cells divide in successive parallel planes perpendicular to their long axis that may lead, in the case of *S. thermophilus*, to elongated cell chains [62].

*W. cibaria* DSM14295 showed a more rectangular shape with visible division septa and a smooth surface (Figure 2e), suggesting a CPS-free cell surface in contrast to the white zones visible in light microscopy. *W. cibaria* is generally able to produce homo- and hetero-polysaccharides [12]. For DSM14295, the CPS synthesis pathway and the binding mechanism to the cell surface are, however, still unknown. As possible reasons for the smoother surface, we assumed that CPS are either attached more loosely to the cells and therefore lost during SEM sample preparation, or CPS exhibited a lower molecular mass compared to CPS from *S. thermophilus* (e.g., DGCC7710 CPS: 1.74 × 10^6^ Da [63]) and were thus less entangled.

It was also possible to distinguish between cells with and without CPS according to their size in SEM images (ST143+: 0.84 ± 0.04 µm; ST143−: 0.75 ± 0.10 µm; cell diameter of their short axis). DSM14295 showed a cell diameter of 0.60 ± 0.04 µm.

### 3.2. Detachment of Capsular Exopolysaccharides from Bacteria Cells

Different thermal, acoustical, and chemical methods for the detachment of CPS from the bacteria cell wall are described in the literature [1,64]. A heat treatment was not appropriate as it resulted in cell lysis, altering cell surface characteristics and interfering with the MATH assay [49]. Chemical treatments with, for example, sodium hydroxide, EDTA, or phenol, may lead to side reactions with cell wall constituents or to polysaccharide hydrolysis [65]. Ultrasonication is a widespread tool, for example, extraction of intracellular substances [66], reduction of yeasts and lactic acid bacteria as a non-thermal alternative [67], modification of polysaccharide properties [68,69], or the enhancement of the emulsification, fermentation, and sensory properties of fermented milk products [70]. This method is fast and easy-to-handle. The energy input can be controlled by amplitude and time, so cell lysis can also be avoided as suggested in the present study. Thus, different ultrasonication conditions were tested (amplitude: 60–100%; time: 10 s–2 min) to determine the lowest energy input necessary for CPS detachment from the cells.

For an amplitude <100%, CPS remained attached to the cells as was confirmed by microscopic images. Increased sonication times at 100% amplitude increased the amount of destroyed cells. The minimum time for removing CPS from the cells was 18 s and thus applied to all further experiments as no cell disruption was detected in the microscopic images (Figure 1b,d,f,h).

**Figure 3:**
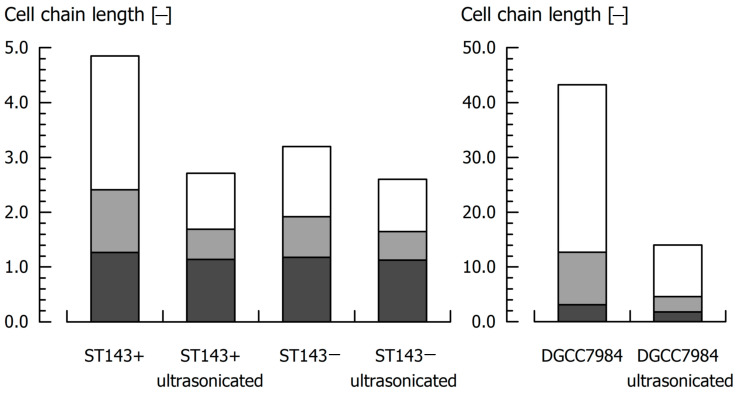
Bacteria cell chain length of *S. thermophilus* before and after ultrasonic treatment for CPS removal. Dark grey: x_10_; light grey: x_50_ (median); white: x_90_ values of the cell chain length distribution.

In the case of *S. thermophilus* strains, ultrasonication also reduced the cell chain length (Figure 3). This effect was most pronounced for ST143+ and DGCC7984, where the cell chains were shortened by 44 and 67% (x_90_), respectively. DGCC7984 showed exceptionally long cell chains (x_90_ = 43.23) compared to ST143− (x_90_ = 3.20), where the treatment had only a small effect (x_90_ = 2.60 after ultrasonication). The shortened cell chains were beneficial for a correct CSH determination, as the tendency of the bacteria cells to sediment during the MATH assay was reduced.

The morphological changes caused by CPS removal were also visible in the SEM images (Figure 2b,d,f). The cell surface of ultrasonicated ST143+ was smoother for the majority of the cells, and appeared similar to that of ST143−, apart from their different state of cell division. This also indicates that the CPS were successfully removed. No damage to the cell surface could be detected, and Z-rings were now clearly visible. No morphological changes were observed for ST143−. This was also true for DSM14295, which was, however, classified as a CPS producer. As already stated, the CPS of the untreated cells may have been lost during SEM sample preparation because of their different attachment to the cell wall compared to *S. thermophilus* cells.

The cell diameter of ST143+ was also significantly reduced by sonication (0.72 ± 0.05 µm). Under the assumption that CPS were not lost during *S. thermophilus* preparation, untreated ST143+ cells exhibited a CPS layer of approx. 120 nm that was one sixth of the cell diameter. For ST143− and DSM14295, no significant changes in the cell diameter (0.76 ± 0.03 and 0.61 ± 0.04 µm, respectively) were observed.

To check that cell viability was not impaired by ultrasonication, fresh medium was inoculated with sonicated cell suspensions (1% (*v*/*v*)) and incubated as stated above. Neither growth kinetics (acidification speed; pH and OD_577_ at the end of cultivation) nor the ability to produce CPS differed from that of cultures with untreated inocula (data not shown).

### 3.3. Influence of Capsular Exopolysaccharides on Cell Surface Hydrophobicity

**Figure 4:**
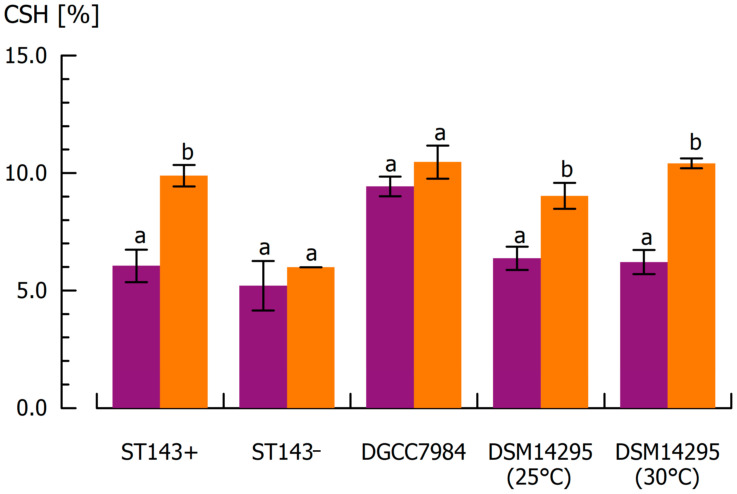
Cell surface hydrophobicity (CSH) of cells from *S. thermophilus* ST143+, ST143−, DGCC7984, and *W. cibaria* DSM14295 before (violet) and after ultrasonic treatment for CPS removal (orange). Mean values within the same strain with different superscripts differed significantly (α = 0.05).

For *S. thermophilus*, no distinction could be made between the CPS positive ST143+ (CSH = 6.0%) and CPS negative ST143− (CSH = 5.2%) (Figure 4). Furthermore, different cultivation temperatures did not affect the hydrophobicity of cells from *W. cibaria* DSM14295. CSH was 6.4% after cultivation at 25 °C and 6.2% after cultivation at 30 °C. CSH of DGCC7984 was highest (9.4%) within the investigated strains. The results underline the strain dependency of cell surface properties, independent from CPS formation, as reported in the literature [39,40]. Particularly for ST143+ and ST143−, which exhibited similar CSH values despite their difference in CPS production, other structural specifics in the cell wall composition must be responsible. For *W. confusa*, a CSH of 27% was reported under similar MATH test conditions, making the strain-dependent differences even more obvious [71].

CSH of ST143+ increased significantly to 9.9% after CPS detachment. This was also observed for DSM14295 independent from cultivation temperatures, whereas the CPS negative strains ST143− and DGCC7984 showed no sonication-induced changes in CSH. This clearly confirms that CPS provide a more hydrophilic character to the cell surface, and thus may have a significant impact on interactions with, for example, milk proteins in fermented dairy systems. Various studies reported that the presence of hydrophilic cell surfaces is associated with CPS, whereas proteins lead to a more hydrophobic surface [11,17,37,38].

**Figure 5:**
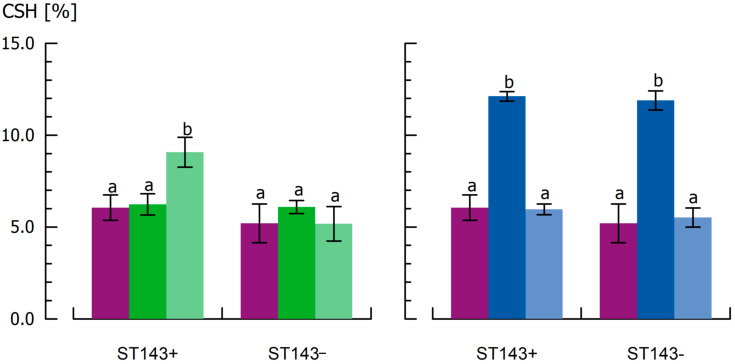
Cell surface hydrophobicity (CSH) of cells from *S. thermophilus* ST143+ and ST143− without further treatment (violet); (**left**) after ultrasonication + subsequent cultivation (dark green) and after ultrasonication + subsequent cultivation + second ultrasonication (light green); (**right**) after freeze drying (dark blue) and after freeze drying + subsequent cultivation (light blue). Mean values within the same strain with different superscripts differ significantly (α = 0.05).

The ultrasonication of the CPS negative strains did not change the CSH, indicating that no destruction of the cells occurred, supporting our findings from SEM images. The MATH assay of re-cultivated, ultrasonicated cells resulted in similar values to those observed in the first assay; CSH was 6.2 and 6.1% for ST143+ and ST143−, respectively, and thereby did not differ significantly from the CSH of untreated cultures (Figure 5, left). The same is true for ultrasonication of the re-cultivated cells: CSH increased for ST143+ (9.1%) due to the detachment of CPS, but did not change significantly for ST143− (5.2%).

Freeze-drying, used for the preparation of cells for moisture sorption analysis, represents a physiological stress situation that, for instance, impairs membrane fluidity [72]. To check whether freeze-drying affects the cell surface properties, the MATH assay was also carried out on freeze-dried cells re-suspended in PBS buffer as well as on freeze-dried and subsequently re-cultivated cells (Figure 5, right). After freeze-drying, the cell surface was much more hydrophobic for both strains (CSH approx. 12%). These findings seem consistent as the complete intra-cellular water was removed by freeze-drying. On the other hand, Selwal et al. [73] found similar CSH before and after freeze-drying of *S. thermophilus* cells. A subsequent fermentation with freeze-dried cells showed similar growth kinetics and CSH as untreated cells.

### 3.4. Influence of Capsular Exopolysaccharides on Moisture Sorption

**Figure 6:**
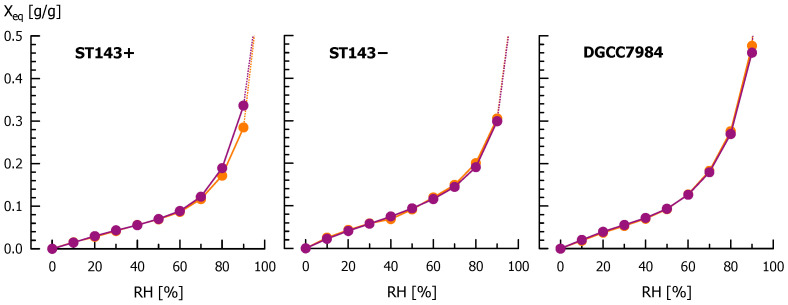
Equilibrium moisture load X_eq_ [g H_2_O/g dry matter] of *S. thermophilus* ST143+, ST143− and DGCC7984 cells at different relative humidity (RH) levels, determined by dynamic vapor sorption analysis. Violet: untreated cells; orange: ultrasonicated cells.

With increasing RH, the moisture sorption isotherms of freeze-dried *S. thermophilus* cells, without and with treatment for CPS removal, showed the sigmoid shape of type II isotherms after Brunauer et al. [74], typical for food and food-related samples and polysaccharides (Figure 6) [75,76,77]. The pronounced increase of equilibrium moisture load (X_eq_) at higher RH is linked to highly hygroscopic substances (e.g., mono- and polysaccharides or salts) [52]. For RH ≤60%, untreated and ultrasonicated cells showed similar adsorption isotherms. At higher RH, the moisture load of untreated cells of ST143+ (X_90%_ = 0.337 ± 0.003 g/g) was 15% higher than for ST143+ without CPS. This indicates that CPS are crucial for moisture adsorption of bacteria cells. For CPS negative strains, the moisture load did not differ significantly between untreated and ultrasonicated cells, as X_90%_ was 0.299 ± 0.007 and 0.305 ± 0.007 g/g (ST143−) and 0.461 ± 0.008 and 0.477 ± 0.010 g/g (DGCC7984), respectively. This supports our previous findings that ultrasonication of CPS negative cells did not change their CSH. The results were confirmed by investigations on a CPS positive *S. thermophilus* DGCC7710. X_90%_ was significantly lower for cells without CPS (CPS were detached from the cells by heat treatment), and X_90%_ for untreated cells was in the same order of magnitude as isolated free exopolysaccharides and CPS of DGCC7710 [63]. However, as for CSH, we suppose that the strain dependent differences in absolute moisture load may be caused by differences in cell wall composition. It can be concluded that the detachment of CPS from the bacteria cells causes an increase in CSH that is accompanied by a reduction of the absorbable moisture.

The moisture load at different RH was also fitted into the GAB equation to obtain the adsorption isotherms (Equation (2); Table 1). At low RH, the cell surface is initially covered with a monolayer of water molecules expressed by X_0_. Although previous studies point to relationships between surface hydrophobicity and X_0_ [78], this was not observed for the investigated *S. thermophilus* strains. The CPS negative strains ST143− and DGCC7984 had higher X_0_ (0.070 and 0.058 g/g, respectively) than ST143+ (0.044 g/g), and were in no case altered by sonication. The k_GAB_ represents the chemical potential of free water (k_GAB_ = 1.0) compared to the water bound in the multilayer of the sample [79]. High k_GAB_ as observed in our study indicated low interactions between the bound water and sample surface. A low C_GAB_ indicated that enthalpy differences between the free and bound water were small. Further dynamic vapor sorption experiments were performed where RH, after achieving mass equilibrium at RH = 0%, was set directly to 98% (without time abort criterion). The time when 50% of the final moisture load X_98%_ was adsorbed (t_1/2_ [min]) can be used to estimate the speed of moisture adsorption. For cells in native state, moisture adsorption was much faster for ST143+ (560 min) compared to ST143− (750 min). This indicates that the transport of water into the cell (ST143−) takes more time than its adsorption at the hygroscopic CPS layer (ST143+). After ultrasonication, both strains showed an accelerated moisture adsorption (520 min for both strains) that may be attributed to the reduced cell chain length and possible changes in the cell wall composition at the former binding sites that were not detected with the previous analyses.

## 4. Conclusions

The aim of this study was to evaluate the influence of CPS on the surface properties of lactic acid bacteria cells. To our knowledge, this is the first time that strain-dependent differences could be eliminated through the approach of using single strains and ultrasonication for CPS detachment. With CPS negative strains, we confirmed that the applied ultrasonication treatment did not affect growth kinetics, cell surface properties (hydrophobicity, moisture load), and morphology of the ultrasonicated cells. Light microscopy and SEM were suitable techniques to qualitatively distinguish between the CPS positive and negative strains. Using RuO_4_ as a staining agent allowed for the visualization of the CPS layer for *S. thermophilus* ST143+, which resulted in a significantly rougher cell surface than cells after CPS removal or CPS negative strains. The thickness of the CPS layer could be determined from the SEM images and was approx. 120 nm. CPS increased the hydrophilic character of the cell surface (reduction of hydrophobicity from approx. 10% to 6%) and led to an increase in speed and the absolute amount of water adsorption of the cells (increase of 15% compared to cells after CPS detachment). This underlines the benefits that could be derived from CPS producing strains regarding, for example, texture, water binding capacity, or syneresis of fermented foods.

## Figures and Tables

**Table 1:** Coefficients of the Guggenheim–Anderson–De Boer (GAB) adsorption isotherms X_0,eq_, C_GAB_, and k_GAB_ for *S. thermophilus* ST143+, ST143−, and DGCC7984 cells without and with ultrasonic treatment for CPS removal.

	X_0,eq_ [g/g]	C_GAB_ [−]	k_GAB_ [−]	r^2^
ST143+	0.044	4.728	0.967	0.999
ST143+ (ultrasonicated)	0.044	4.875	0.944	0.999
ST143−	0.058	6.881	0.900	0.999
ST143− (ultrasonicated)	0.060	6.202	0.899	0.999
DGCC7984	0.070	3.056	0.952	0.999
DGCC7984 (ultrasonicated)	0.073	2.505	0.952	0.999
